# hsa_circRNA6448-14 promotes carcinogenesis in esophageal squamous cell carcinoma

**DOI:** 10.18632/aging.103650

**Published:** 2020-08-15

**Authors:** Yaowen Zhang, Xiang Yuan, Ning Yue, Lidong Wang, Junqi Liu, Ningtao Dai, Haijun Yang, Ruitai Fan, Fuyou Zhou

**Affiliations:** 1 Department of Radiation Oncology, Anyang Cancer Hospital, Anyang 455000, China; 2 Department of Radiation Oncology, Henan Key Laboratory for Cancer Research, The first Affiliated Hospital of Zhengzhou University, Zhengzhou 450000, China; 3 Henan Key Laboratory of Cancer Epigenetics, Cancer Institute, The First Affiliated Hospital and College of Clinical Medicine of Henan University of Science and Technology, Luoyang 471000, China; 4 Department of Radiation Oncology, Rutgers - Cancer Institute of New Jersey, Rutgers - Robert Wood Johnson Medical School, New Brunswick, NJ 08903, USA

**Keywords:** hsa_circRNA6448-14, esophageal squamous cell carcinoma, microarray, proliferation, microRNA sponge

## Abstract

Circular RNAs (circRNAs) play important roles in cancer progression. hsa_circRNA6448-14 originates from exon 5 to exon 11 of the TGFBI gene. We investigated the roles of hsa_circRNA6448-14 in esophageal squamous cell carcinoma (ESCC) with microarrays and quantitative real-time polymerase chain reaction (qRT-PCR), Kaplan-Meier analysis, loss-of-function and gain-of-function assays, and pull-down assays for miRNA binding. The hsa_circRNA6448-14-miRNA-mRNA network was drawn using Circos. hsa_circRNA6448-14 was significantly upregulated in ESCC tissues and cell lines. As a diagnostic biomarker, hsa_circRNA6448-14 had an area under the curve (AUC), sensitivity, and specificity of 0.906, 82.9%, and 85.5%, respectively. hsa_circRNA6448-14 upregulation was correlated with poor differentiation, advanced pTNM stage, poor disease-free survival (DFS), and poor overall survival (OS). Elevated hsa_circRNA6448-14 promoted cell proliferation, migration, invasion, and inhibited apoptosis in vitro. hsa_circRNA6448-14 functioned as a miRNA sponge to competitively bind miR-455-3p, and hsa_circRNA6448-14 expression negatively correlated with that of miR-455-3p. hsa_circRNA6448-14 promoted carcinogenesis in ESCC, suggesting that hsa_circRNA6448-14 could serve as a diagnostic and prognostic biomarker for ESCC.

## INTRODUCTION

Esophageal cancer (EC) is one of the most frequently occurring malignant neoplasms worldwide, with high incidence and mortality [1, 2]. It is characterized by international differences in incidence and pathology [3, 4]. Esophageal squamous cell carcinoma (ESCC) accounts for more than 90% of all EC in China. The highest incidence in China is found in the Taihang mountain region, Henan province [5]. Due to local recurrence and distant metastasis, ESCC has a poor prognosis, with a 5-year survival rate of less than 15-20% [6]. Since early detection plays a key role in controlling the mortality of ESCC, it is urgent to identify novel sensitive and effective biomarkers for ESCC.

Circular RNAs (circRNAs) are a novel class of non-coding RNAs with a covalently closed circular structure, formed by back-splicing, different from canonical splicing of linear RNAs. In 1976, Sanger et al. [7] discovered the first circRNA in a plant RNA virus. In 1979, circRNA was observed in the cytoplasm of eukaryotes [8]. In 1991, Nigro et al. [9] confirmed that circRNA existed in human cells. However, circRNAs were initially considered junk generated in aberrant splicing events [10]. More recently, with deep RNA sequencing and bioinformatics, thousands of circRNAs have been identified in eukaryotes. They can be evolutionarily conserved [11] and show tissue-specific expression and roles in pathological conditions [12]. They are regulated by specific cis-elements and trans-factors [13], indicating they are functional [14]. circRNAs have been implicated in several human disorders including cancer [15–20].

Previous studies demonstrated that circRNAs have diverse biological functions, including acting as miRNA or protein sponges [13, 21]. circRNAs may act as oncogenes or tumor-suppressors [22, 23]. circRNAs may be more stable than linear RNAs [13] as biomarkers. Previous studies confirmed circRNAs are biomarkers for cancers including prostate [24], liver [25], gastric [26], and lung [27]. However, the function of circRNAs in ESCC was unclear.

Using circRNA microarray profiling, we identified hsa_circRNA6448-14 as a circRNA that was significantly up-regulated in ESCC tissues and cell lines. High expression of hsa_circRNA6448-14 was associated with poor differentiation, higher pTNM stage, and poor prognosis in ESCC patients. In vitro studies suggested that hsa_circRNA6448-14 promoted cell proliferation, migration, and invasion, and acted as a miR-455-3p sponge. Our study indicated that hsa_circRNA6448-14 promotes the progression of ESCC.

## RESULTS

### circRNA expression profiles in ESCC and adjacent normal tissues by microarray analysis

**Figure 1:**
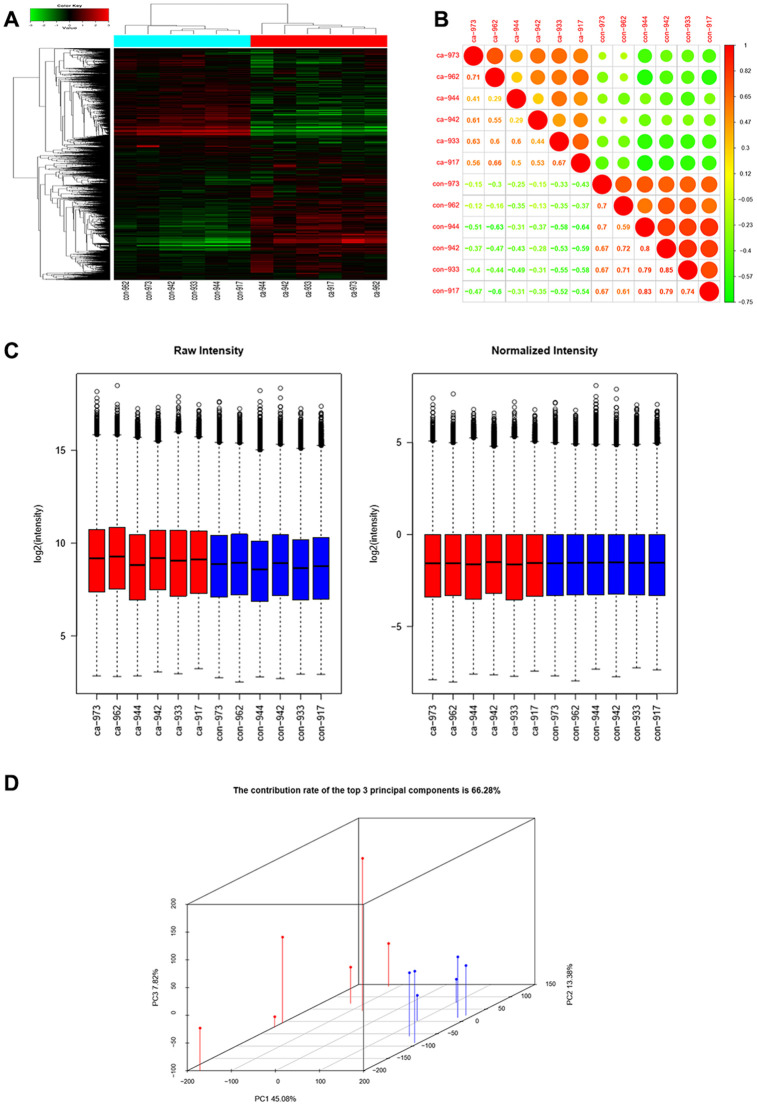
**CircRNAs expression profiles in ESCC and adjacent normal tissues.** (**A**) Hierarchical clustering showed that a large number of circRNAs were expressed in two groups. (**B**) Correlation analysis showed a high correlation of samples. (**C**) Box plots showed that samples had comparable intensities. (**D**) Three-dimensional principal component analysis (PCA 3D) indicated high similarity of samples.

**Figure 2:**
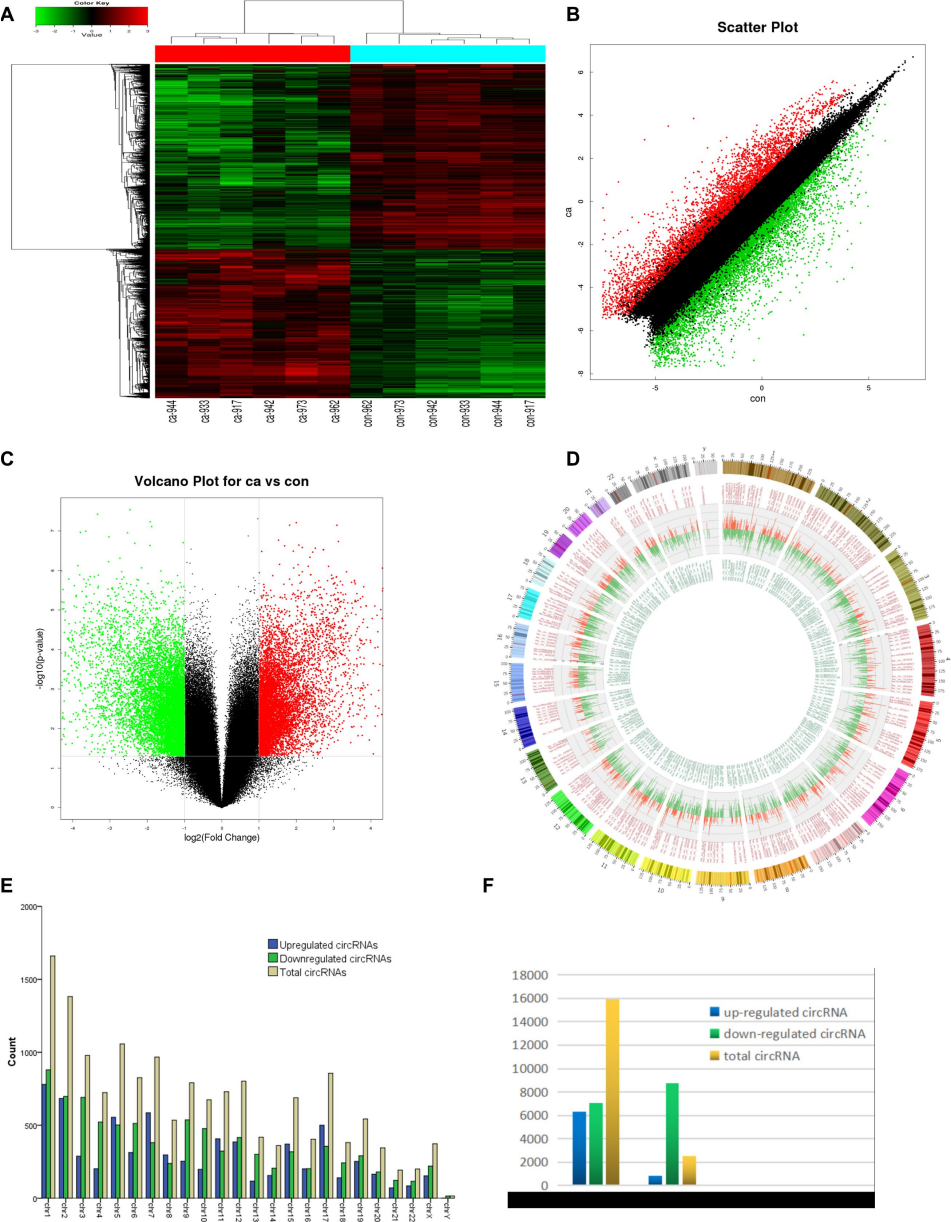
**circRNA expression profile differences between ESCC and paired adjacent normal tissues.** (**A**) Hierarchical clustering of circRNA expression profiles across samples. Each column is a tissue sample, and each row is a circRNA. (**B**) Scatter plot of differences in circRNA expression in ESCC and normal tissues. (**C**) Volcano plot showing dysregulated circRNAs. (**D**) Circos plot indicating differentially expressed circRNAs. Column width represents the number of differentially expressed circRNAs, and the column length the degree of differential expression. (**E**) Chromosomal distributions of differentially expressed circRNAs. Blue, green, and yellow represent upregulated, downregulated, and total circRNAs, respectively. (**F**) CircRNA source: 84.02% of dysregulated circRNAs originated from exons. Ca=ESCC tissues; Con=adjacent normal tissues. Red and green indicate upregulated and downregulated circRNAs, respectively.

Six pairs of ESCC and adjacent normal tissues were randomly selected for circRNA microarray analysis. A total of 149789 circRNAs were detected. Cluster analysis indicated that a large number were expressed in two groups; correlation analysis indicated a high correlation of samples; box plot analysis showed that samples had comparable intensities; and a three-dimensional principal component analysis (PCA 3D) indicated high similarity between samples (Figure 1). A total of 15908 circRNAs were significantly dysregulated between ESCC and matched normal tissues after filtering. Among them, 7161 circRNAs (45.01%) were upregulated and 8747 (54.98%) were downregulated. Hierarchical clustering analysis, scatter plot, volcano plot, and Circos plot clearly showed differential circRNA expression between cancer and non-cancer groups (Figure 2A-2D). The chromosomal distribution of differentially expressed circRNAs showed that most were transcribed from chr1, chr2, chr3, chr5, chr7, and chr17, with a few transcribed from chr21, chr22, chrX, or chrY (Figure 2E). Most differentially expressed circRNAs (84.02%) originated from exons (Figure 2F).

### qRT-PCR validation of circRNA expression profiles

**Figure 3:**
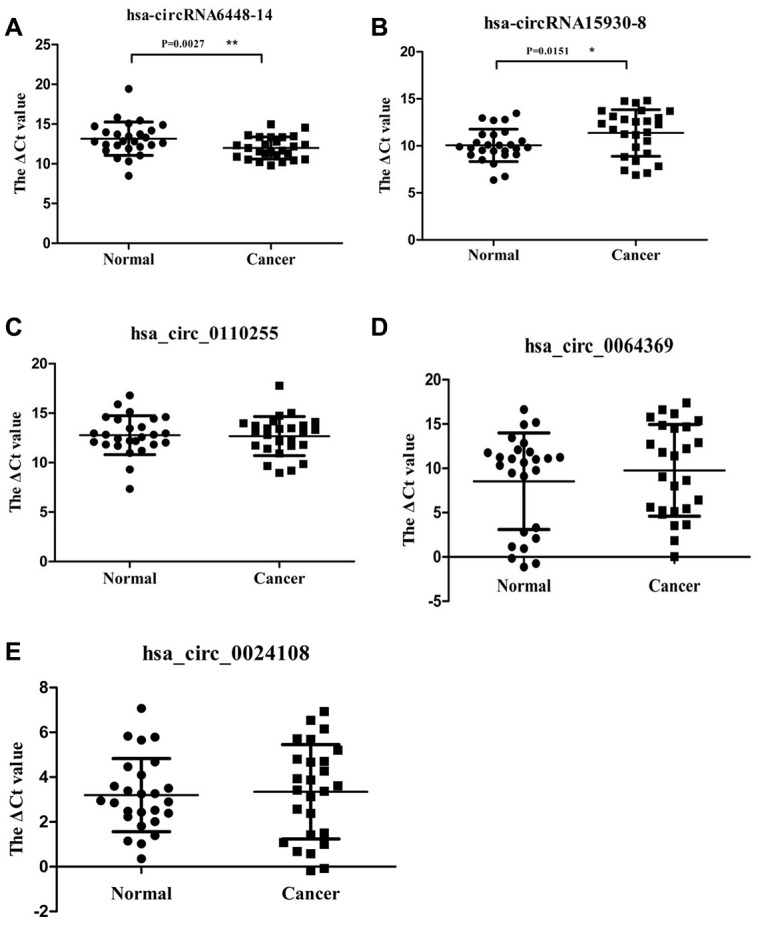
**Relative expression of 5 circRNAs verified by qRT-PCR in 26 pairs of ESCC and adjacent normal samples.** (**A**) hsa-circRNA6448-14. (**B**) hsa-circRNA15930-8. (**C**) hsa_circ_0110255. (**D**) hsa_circ_0064369. (**E**) hsa_circ_0024108. * p < 0.05, ** p < 0.01.

Candidate clinically relevant biomarkers from the microarray results were selected from upregulated and downregulated circRNAs according to the following criteria: 1) fold change, p value, and moderate microarray signal value; 2) more than two miRNA binding sites; 3) predicted target miRNAs associated with ESCC. Based on these criteria, five circRNAs were selected for further validation (Table 1). They were validated using qRT-PCR in 26 pairs of ESCC and adjacent normal tissues. The dissolution curve showed that all the five circRNAs had single peaks and the peak values were high, indicating that the specificity of PCR amplification primers were good (Supplementary Figure 1). hsa_circRNA6448-14 was significantly up-regulated in ESCC tissues compared to adjacent normal tissues (p < 0.05), hsa_circRNA15930-8 was significantly down-regulated (p < 0.05), and hsa_circ_0110255, hsa_circ_0064369, and hsa_circ_0024108 showed no significant differences (p > 0.05) (Figure 3).

**Table 1:** Five candidate circRNAs selected for qRT-PCR validation.

**CircRNA**	**P**	**FC**	**Regulated**	**Chr**	**Source**	**Host gene**
hsa-circRNA6448-14	0.003	23.95	up	chr5	exon	TGFBI
hsa_circ_0110255	0.000*	195.3	up	chr1	exon	COL11A1
hsa-circRNA15930-8	0.000*	16.81	down	chr9	exon	SH2D3C
hsa_circ_0064369	0.000*	8.44	down	chr3	exon	TMEM40
hsa_circ_0024108	0.000*	330.3	up	chr11	exon	MMP1

*p < 0.001. FC=fold change.

### hsa_circRNA6448-14 as a diagnostic biomarker in ESCC

**Figure 4:**
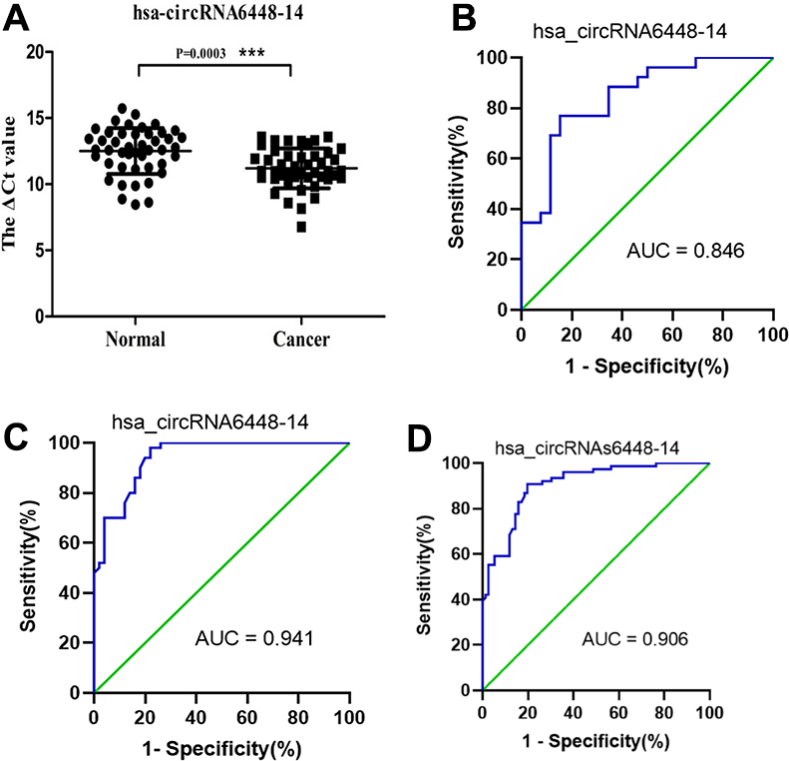
**hsa_circRNA6448-14 validation as a diagnostic biomarker for ESCC.** (**A**) Expression of hsa_circRNA6448-14 determined by qRT-PCR, relative to GAPDH (n=50). (**B**) ROC curve analyses for hsa_circRNA6448-14 in ESCC, n=26; (**C**) n=50; and (**D**) n=76.

ROC curve analysis was performed to determine the diagnostic values of hsa_circRNA6448-14 and hsa_circRNA15930-8 for ESCC. The area under the curve (AUC), sensitivity, and specificity of hsa_circRNA6448-14 were 0.846 (95% CI: 0.738-0.954, p = 0.045), 80.8%, and 77%; for hsa_circRNA15930-8 the values were 0.673 (95% CI: 0.519-0.828, p = 0.032), 73.1%, and 50.0%, respectively, for the diagnosis of ESCC. Because hsa_circRNA6448-14 showed a greater AUC and lower p value than hsa_circRNA15930-8, the former was chosen for validation in more samples and we expanded the sample size to 50 pairs. hsa_circRNA6448-14 displayed significant upregulation in ESCC tissues compared with adjacent normal tissues (p < 0.05), confirming the microarray analysis and the first qRT-PCR validation set and indicating the reliability of these results (Figure 4). ROC curves on the additional 50 samples gave an AUC of 0.941 (95% CI: 0.893-0.990, p = 0.023), with sensitivity and specificity of 84.6% and 86.0%, respectively. The 76 patients were pooled and analyzed, which confirmed that hsa_circRNA6448-14 was significantly up-regulated in ESCC, and ROC curves showed AUC, sensitivity, and specificity of 0.906 (95% CI: 0.899-0.903, p = 0.021), 82.9%, and 85.5%.

### hsa_circRNA6448-14 as a prognostic biomarker in ESCC

**Figure 5:**
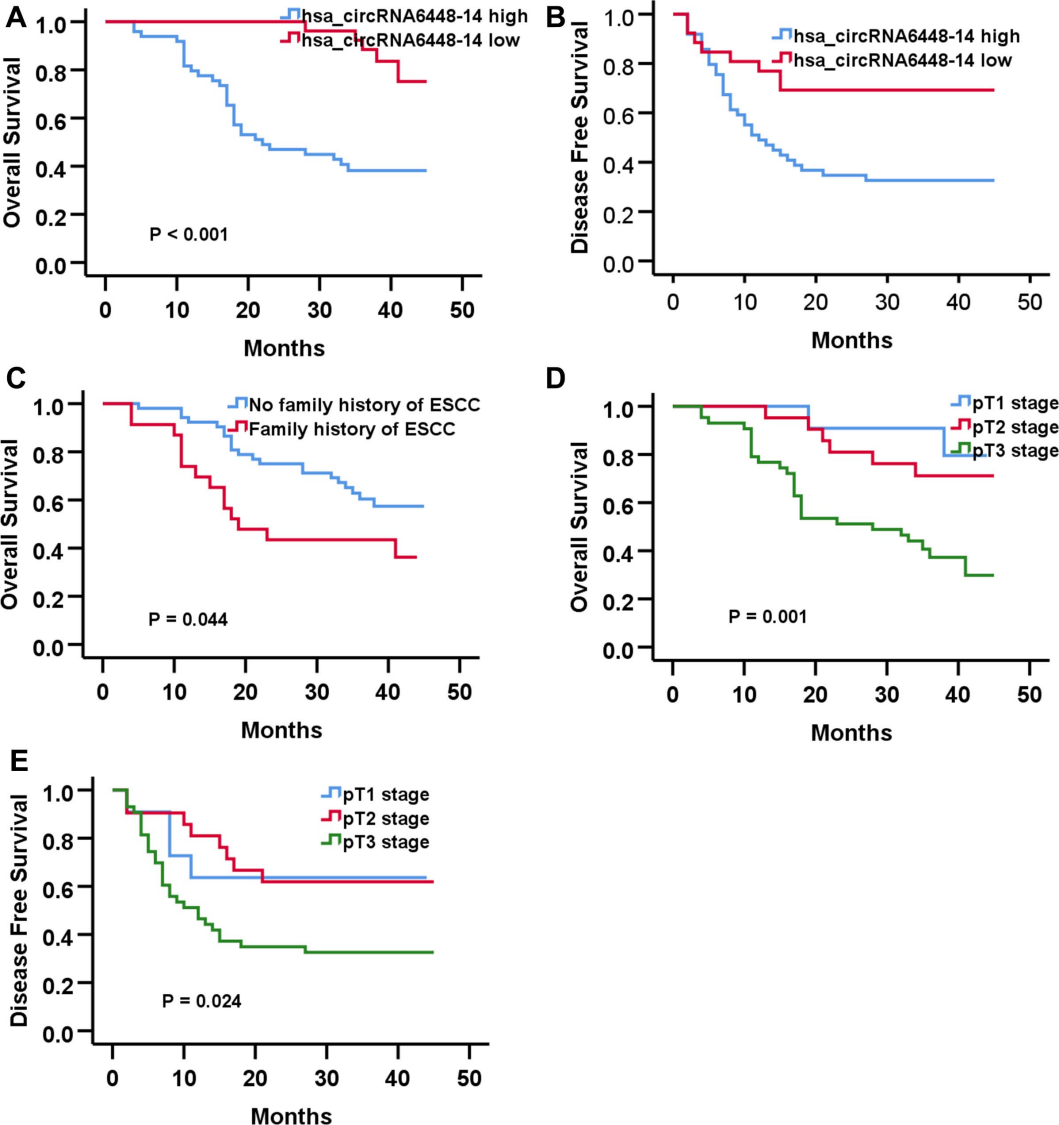
**Prognostic significance of hsa-circRNA6448-14 in 76 case of ESS by Kaplan-Meier analyses.** (**A**) OS (**B**) DFS (**C**) OS based on family history of ESCC. (**D**) OS based on pT stage. (**E**) DFS based on pT stage.

The median hsa_circRNA6448-14 expression of all cases was chosen as the cutoff for dividing the dataset into high and low expression groups. Analysis of clinical pathological data showed high expression of hsa_circRNA6448-14 was positively associated with differentiation and pTNM stage (Table 2). All patients were followed-up until December 25, 2019. The follow-up time was 4 - 45 months (median: 37 months), and the follow-up rate was 100%. The 1, 2 and 3 year OS was 85%, 65%, and 55%, and the DFS was 57%, 47%, 45% for the 76 patients. Kaplan-Meier analysis revealed that ESCC patients with hsa_circRNA6448-14 high expression had shorter OS and DFS than those with hsa_circRNA6448-14 low expression. Univariate and Cox multivariate analysis indicated that pT stage and hsa_circRNA6448-14 expression were independent prognostic factors for postoperative DFS in ESCC (p = 0.031, p = 0.014), with HR values of 1.755 (95% CI = 1.054-2.921) and 0.376 (95% CI = 0.173-0.819), respectively. Family history of ESCC, pT stage, and hsa_circRNA6448-14 expression were independent prognostic factors for postoperative OS in ESCC (p = 0.033, p = 0.007, p = 0.003), with HR values of 2.149 (95% CI = 1.062 - 4.347), 2.516 (95% CI = 1.280 - 4.947) and 0.199 (95% CI = 0.069 - 0.573), respectively. hsa_circRNA6448-14 was identified as a potential prognostic biomarker in ESCC (Figure 5, Table 3).

**Table 2:** Correlation between hsa_circRNA6448-14 expression and clinical/pathological characteristics in ESCC.

**Characteristic**		**Number**	**hsa_circRNA6448-14 expression**		**χ^2^**	**P**
			**high**	**low**		
Gender	Male	46	31	15	0.133	0.715
	Female	30	19	11		
Age (years)	> 65	33	24	9	1.247	0.332
	≤ 65	43	26	17		
Tumor location	Upper	6	5	1	5.211	0.070
	Middle	53	38	15		
	Lower	17	7	10		
Smoking	No	38	24	14	0.234	0.629
	Yes	38	26	12		
Drinking	No	44	28	16	0.215	0.643
	Yes	32	22	10		
Family history	No	53	32	21	2.279	0.131
	Yes	23	18	5		
Tumor size	> 4 cm	28	19	9	0.084	0.772
	≤ 4 cm	48	31	17		
Differentiation	Well	15	6	9	11.171	0.003
	Moderate	43	27	16		
	Poor	18	17	1		
pT stage	pT1	11	6	5	1.816	0.403
	pT2	22	13	9		
	pT3	43	31	12		
pN stage	pN0	40	25	15	0.418	0.793
	pN1	26	18	8		
	pN3	10	7	3		
pTNM stage	I-II	43	24	19	4.378	0.036
	III-IV	33	26	7		
Total lymph node dissection (n)	≥15	45	26	19	3.146	0.076
	<15	31	24	7		

**Table 3:** Univariate and multivariate analysis for postoperative OS and DFS in 76 cases of ESCC.

**Factor**	**OS**				
	**Univariate**			**Multivariate**	
	**χ^2^**	**P value**	**P value**	**Exp(B)**	**95%CI**
Family history	4.392	0.036	0.033	2.149	1.062-4.347
Differentiation	7.587	0.023	0.504	1.223	0.677-2.210
pT stage	13.049	0.001	0.007	2.516	1.280-4.947
pN stage	7.293	0.026	0.895	1.056	0.471-2.369
pTNM stage	13.345	0.000	0.444	1.547	0.506-4.729
hsa_circRNA644814 expression	15.553	0.000	0.003	0.199	0.069-0.573
	DFS				
pT stage	7.427	0.024	0.031	1.755	1.054-2.921
hsa_circRNA644814 expression	7.599	0.006	0.014	0.376	0.173-0.819

### hsa_circRNA6448-14 promotes ESCC cell progression

**Figure 6:**
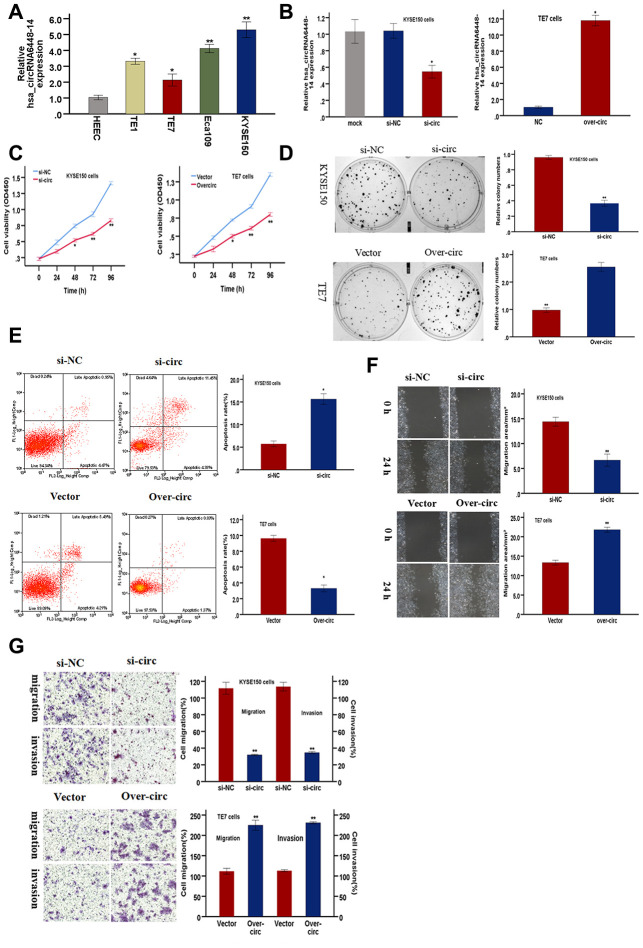
**hsa-circRNA6448-14 promotes ESCC progression in cell lines.** (**A**) Relative expression of hsa-circRNA6448-14 in ESCC lines and normal cell line by qRT-PCR. (**B**) hsa-circRNA6448-14 expression detected after transfection in KYSE-150 and TE-7 cells. (**C**) CCK-8 assays to determine viability of KYSE150 and TE7 cells. (**D**) Colony-formation assays to measure proliferation after hsa_circRNA6448-14 knockdown in KYSE-150 cells or overexpression in TE7cells. (**E**) Flow cytometry to detect apoptosis of KYSE150 and TE7 cells after transfection. (**F**) Wound healing assays to detect cell migration capacities of KYSE150 and TE7 cells after transfection. (**G**) Transwell assays to detect cell migration and invasion capacities of KYSE150 and TE7 cells after transfection. *p < 0.05, **p <0.01.

Functional assays were conducted to validate the role of hsa_circRNA6448-14 in ESCC progression. hsa_circRNA6448-14 expression was detected in four ESCC cell lines and one normal human esophageal epithelial cell line (HEEC) by qRT-PCR. hsa_circRNA6448-14 was elevated in ESCC cell lines compared to HEEC (Figure 6A). hsa_circRNA6448-14 expression was highest in KYSE150 cells and lowest in TE7 cells, so these two cell lines were respectively selected for hsa_circRNA6448-14 knockdown and overexpression. Expression of hsa_circRNA6448-14 was effectively silenced by si-circRNA6448-1 (si-circ) in KYSE150 cells, and significantly up-regulated by overexpression (over-circ) in TE7 cells (Figure 6B). A CCK-8 assay revealed that si-circ significantly suppressed cell growth, and over-circ promoted cell viability (Figure 6C). A colony formation assay confirmed that si-circ inhibited proliferation, while over-circ promoted it (Figure 6D). Apoptosis, measured by flow cytometry, was significantly promoted by si-circ, and decreased by over-circ (Figure 6E). Wound healing and Transwell assays revealed that si-circ significantly decreased migration; over-circ increased migration (Figure 6F, Figure 6G). Invasion, as measured by Transwell assays with Matrigel, showed si-circ reduced and over-circ enhanced invasion (Figure 6G).

### hsa_circRNA6448-14 targeted a miRNA-mRNA network and served as a sponge for miR-455-3p

**Figure 7:**
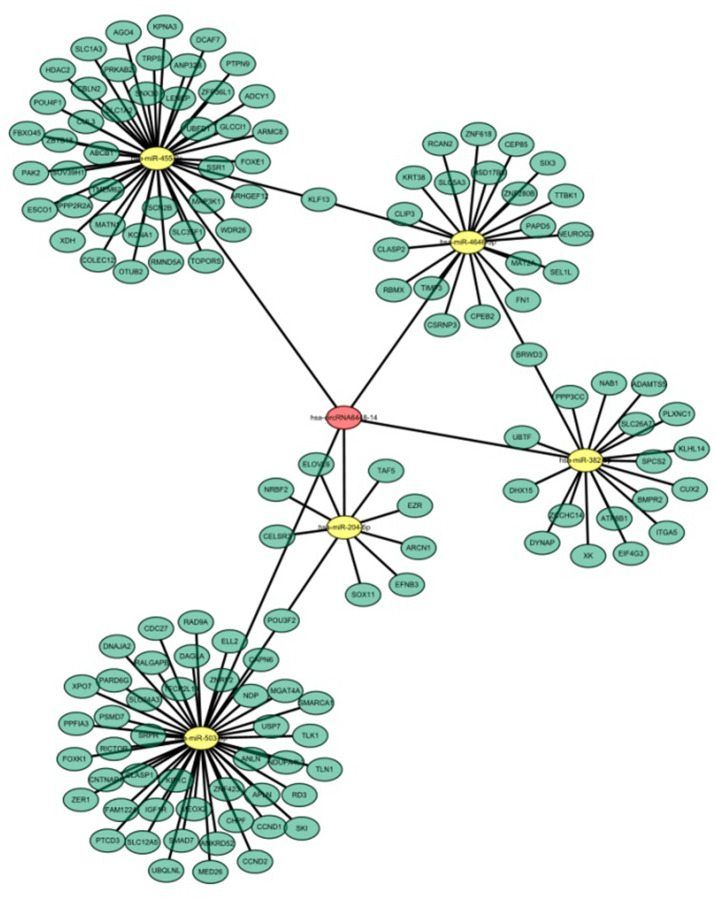
**hsa_circRNA6448-14-miRNA-mRNA network in ESCC.**

Recent evidence shows that many circRNAs may function as miRNA sponges [12]. The genomic locus of hsa_circRNA6448-14 is on chromosome 5, and the predicted gene sequence of its transcript is ENST00000442011. We identified and ranked the putative target miRNAs of hsa_circRNA6448-14 based on mirSVR scoring. Five miRNAs were found with overlapping results. We hypothesized that hsa_circRNA6448-14 acted as a miRNA sponge to regulate this circRNA-miRNA-mRNA network, and that the interactions could be predicted by TargetScan and miRanda. A total of five miRNAs and 138 mRNAs were predicted to interact with hsa_circRNA6448-14. The possible miRNA binding sites of hsa_circRNA6448-14 were predicted to be miR-503-5p, miR-455-3p, miR-4646-5p, miR-382-5p and miR-204-5p, and the hsa_circRNA6448-14-miRNA-mRNA network was constructed (Figure 7). GO and KEGG pathway analysis was performed. GO annotation analysis involved three major categories: biological processes, cell component, and molecular function. hsa_circRNA6448-14 showed a strong relationship with biological processes including cell processes, single-organism processes, and metabolic processes. The cell, cell part, and organelle were identified as the cell components. In the molecular function classification, binding was the most prominent category, followed by catalytic activity and nucleic acid binding transcription factor activity (Supplementary Figure 2A). KEGG pathway analysis showed that hsa_circRNA6448-14 may participate in the regulation of axonal guidance, catalytic activity, adhesion plaques, and tumor proteoglycan pathways (Supplementary Figure 2B). Analysis using publicly available algorithms (Target Scan, miRWalk, and miRanda) showed that a number of negative regulators, including RICTOR, SKI, NDP, SMAD7, IGF1R, CCND1, RAD9A, TIMP3, FN1, XOH, PTPN9, ABCB1, ADCY1, CUL3, SUV39H1, ITGA5, and EZR were associated with ESCC.

**Figure 8:**
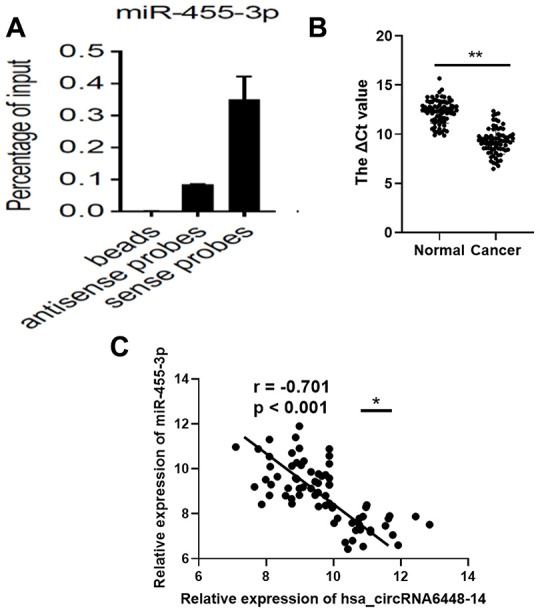
**miR-455-3p in ESCC tissues.** (**A**) RNA pull-down array to detect the enrichment of hsa_circRNA6448-14 and miR-455-3p in ESCC tissues. (**B**) Expression of miR-455-3p in 76 cases of ESCC tissues and adjacent normal tissues (** p < 0.01). (**C**) Correlation analysis of expression between hsa_circRNA6448-14 and miR-455-3p in 76 cases of ESCC (* p < 0.05).

To validate binding of the miRNAs to hsa_circRNA6448-14, we conducted RNA pull down assays using biotin-labeled probes targeting the hsa_circRNA6448-14 junction site (Supplementary Figure 3). Based on the hsa_circRNA6448-14-miRNA-mRNA network, we selected two miRNAs, miR-455-3p and miR-204-5p for pulldown assays. The results showed that hsa_circRNA6448-14 could bind to miR-455-3p, but not miR-204-5p (Figure 8A). Furthermore, we analyzed the expression of miR-455-3p in 76 pairs of ESCC tissues, and determined that the expression of miR-455-3p was significantly down-regulated in ESCC tissues compared to adjacent normal tissues (Figure 8B). The expression of miR-455-3p was negatively correlated with that of hsa_circRNA6448-14 in 76 pairs of ESCC tissues (r = -0.701, p < 0.001, Figure 8C).

## DISCUSSION

Recently, circRNAs have been implicated in cancer occurrence and development. circRNA expression profiling is a prerequisite to identify novel tumor suppressors and oncogenic circRNAs, and to elucidate their mechanisms and functions [28]. In this study, we selected ESCC patients from a high incidence area and compared the circRNA profiles of ESCC and adjacent normal tissues by RNA-seq. Through hierarchical clustering analysis of differentially expressed circRNAs, samples separated into cancer and non-cancer groups, confirming differential expression patterns of circRNAs in ESCC and adjacent normal tissues. These data strongly suggested that circRNAs were involved in the pathogenesis of ESCC.

A total of 15908 significantly dysregulated circRNAs in ESCC were identified, predominantly transcribed from chr1 and chr2, suggesting that the expression patterns may be related to certain biological processes. circRNA sequences largely determine their binding targets. circRNAs can be exonic, intronic, or exonic-intronic, although more than 80% are derived from exons [29, 30]. In our study, the majority of differentially expressed circRNAs were exonic (84.02%). The proportion of up-regulated and down-regulated circRNAs was 88.41% and 80.43%, respectively, consistent with previous reports [30, 31].

Based on RNA-seq and qRT-PCR validation, we identified a circRNA up-regulated in ESCC that was identified in the authoritative database DeepBase as hsa_circRNA6448-14. hsa_circRNA6448-14 had not been previously reported in ESCC, and so was identified as a candidate oncogene in ESCC for the first time. Most circRNAs are expressed from known protein-coding genes and consist of a single or multiple exons [12]. hsa_circRNA6448-14 originates from exon 5 to exon 11 of the TGFBI gene located on chr5, and information is available about it (Supplementary Table 3).

The expression and characteristics of circRNAs, including tissue/cell specificity and stability, make them ideal biomarkers. Several circRNAs have been reported as biomarkers in ESCC. hsa_circ_0001946 is downregulated in ESCC; its overexpression can reduce cell proliferation, migration, and invasion, and predict recurrence, OS, and DFS in ESCC [31]. hsa_circ_0006948 is overexpressed in ESCC; high hsa_circ_0006948 was positively correlated with lymph node metastasis and poor prognosis [32]. cirs-7 is significantly up-regulated in ESCC tissues compared to normal; ESCC patients with cirs-7 overexpression had poor OS [33]. We found that hsa_circRNA6448-14 was more abundant in ESCC, and distinguished ESCC from normal tissues as a diagnostic biomarker. hsa_circRNA6448-14 was positively correlated with differentiation and pTNM stage. Patients with high hsa_circRNA6448-14 expression had poor DFS and OS. These results suggest that hsa_circRNA6448-14 expression could provide diagnostic and prognostic value for ESCC patients.

Loss-of-function and gain-of-function experiments in vitro demonstrated that hsa_circRNA6448-14 was associated with proliferation, migration, invasion, and apoptosis. These results strongly suggest that hsa_circRNA6448-14 plays a role in the progression of ESCC.

Emerging evidence confirms that circRNA acts to competitively bind with miRNAs [34]. For instance, circCDR1as harbors over 70 conserved binding sites and acts as a sponge for miR-7, regulating the expression of human epidermal growth factor receptor and others [35].

We constructed a hsa_circRNA6448-14-miRNA-mRNA network with predicted functions and mechanisms by GO and Pathway analysis. RNA pulldown experiments confirmed that hsa_circRNA6448-14 targeted miR-455-3p in ESCC. miR-455-3p has been demonstrated to play a role in tumorigenesis and be associated with HCC [36, 37]. miR-455-3p, STK17B, and the AKT/GSK-3β/Snail pathway may operate in combination to regulate epithelial-mesenchymal transition (EMT) and metastasis in HCC [38]. miR-455-3p mediates GATA3 tumor suppression in mammary epithelial cells by inhibiting TGF-ß signaling [39]. Downregulation of miRNA-455-3p links proliferation and drug resistance of pancreatic cancer cells by targeting TAZ [40]. These results indicate a pro-oncogenic role of miR-455-3p in tumor progression. miR-455-3p acts as a prognostic marker and inhibits the proliferation and invasion of ESCC by targeting FAM83F [41]. We found downregulated miR455-3p in ESCC tissues, and a negative correlation between miR-455-3p and hsa_circRNA6448-14 expression, suggesting that hsa_circRNA6448-14 affects ESCC progression by negatively regulating miR-455-3p. miRNAs bind to the 3’UTR of mRNA and inhibit gene functions [42]. We predicted potential mRNA binding sites for miR-455-3p, and will study their regulatory mechanisms in depth.

In conclusion, this study identified a profile of dysregulated circRNAs in ESCC in patients from a high incidence area of China. We found that hsa_circRNA6448-14 was upregulated in ESCC tissues and cell lines, and may predict diagnosis and clinical outcomes. hsa_circRNA6448-14 may promote ESCC carcinogenesis by decreasing miR-455-3p. Additional rigorous clinical and fundamental studies are needed to confirm the present results.

## MATERIALS AND METHODS

### Patients and cell lines

The 82 pairs of freshly frozen ESCC and adjacent normal tissues were obtained from patients who underwent operations at the Anyang Cancer Hospital (Henan, China) between June 2015 and April 2016. All patients were from high incidence areas in the Taihang Mountain of China, and received no radiotherapy, chemotherapy, or targeted therapy before surgery. The patients included 49 males and 33 females; ages ranged from 45 to 77 years, with a median of 61 years. Tumor stages were determined according to the eighth edition of the American Joint Committee on Cancer tumor-node-metastasis (TNM) staging criteria [42]. Six pairs of samples were used for microarray profiling, and the remaining 76 pairs of samples were used for validation by quantitative real-time polymerase chain reaction (qRT-PCR). The study was approved by the Ethics Committee of the Anyang Cancer Hospital, and all patients provided written informed consent.

ESCC cell lines including TE1, TE7, Eca109, KYSE150, and human normal esophageal epithelial cells HEEC were obtained from Cell Bank of the Chinese Academy of Sciences (Shanghai).

### CircRNA microarray

Microarrays (Capitalbiotech, human circRNA Array v2., Beijing, China) were performed in six pairs of ESCC and adjacent normal tissues. RNA digestion, amplification, and labeling were performed according to the protocol provided. A total RNA extraction kit (Capitalbiotech, Beijing, China) was used. RNA was resuspended in RNase-free water. RNA integrity was assessed using standard denaturing agarose gel electrophoresis. RNA purity and concentration were determined by OD260/280 readings using a ND1000 spectrophotometer (NanoDrop, Wilmington, DE, United States). RNA was stored at -80°C until use. The circRNA array data were summarized, normalized, and subjected to quality control using GeneSpring software V13.0 (Agilent). The differentially expressed circRNAs were selected according to threshold values of ≥ 2 and ≤ −2 -fold change (FC) and p value of < 0.05.

### qRT-PCR

Total RNA samples were reverse-transcribed (RT) into complementary DNA (cDNA) with a random primer using a PrimeScript RT reagent kit with gRNA Eraser according to the manufacturer’s protocols (Takara Bio, Nojihigashi, Kusatsu, Japan). qRT-PCR was performed using SYBR-Green Premix Ex Taq (Takara Bio, Nojihigashi, Kusatsu, Japan) and monitored on an ABI PRISM 7500 Sequence Detection System (Applied Biosystems, Life Technologies, Waltham, MA, USA). Glyceraldehyde-3-phosphate dehydrogenase (GAPDH) was used as a reference gene. The primer sequences are listed in Supplementary Table 1. The appearance of a single peak in the melting curve analysis indicated primer specificity. All of the experiments were performed in triplicate and data were analyzed using the 2^-ΔΔCT^ method to establish the relative expression levels of circRNAs.

### Cell culture and cell transfection

Vectors for overexpression of hsa_circRNA6448-14 and si-hsa_circRNA6448-14 and their corresponding controls were commercially obtained from Gene Company (Shanghai, China). Scrambled sequences and empty vector were used as negative controls. The hsa_circRNA6448-14 siRNA and overexpression plasmid were transfected into KYSE150 cells and TE7 cells respectively using Lipofectamine 2000 (Invitrogen), according to the instructions, and harvested for experiment after 48 hours. The transfection efficiency was detected by qRT-PCR.

### Cell proliferation assays

Details of cell counting Kit-8 (CCK-8) and colony formation assays are available in previous studies [43].

### Wound healing assay

Cells transfected for 24 hours were seeded in 6-well plates at a 3×10^5^ cells per well and cultured at 37°C for 12 hours. A 10 μL pipette tip was used to draw a linear wound in the middle of each well, and the cells were further cultured for 24 hours after washing. Photos were taken at zero and 24 hours after scrapping.

### Transwell assay

Cell invasion and migration assays were carried out using Transwell chambers (8-mm pore size, Corning) pre-coated with (for invasion assay) or without Matrigel (for migration assay) (BD Pharmingen, USA). Cells were serum starved for 24 hours, then seeded in the upper chamber with 200 ml of FBS-free medium, while 600 ml complete medium with 10% FBS was added to the lower chamber as a chemoattractant. After incubation for 24 hours, non-migrating or non-invading cells were removed from the upper chamber. Cells that passed through the membrane were fixed with 4% paraformaldehyde for 30 minutes and stained with 0.1% crystal violet solution (Sigma, St. Louis, USA) for 30 minutes. Cells were counted under a light microscope (Leica, German). All experiments were performed in triplicate.

### Apoptosis assay

Transfected cells were collected and washed with PBS and analyzed with Annexin V: PI apoptosis detection kit (BD Bioscience, USA). Cells were resuspended in the provided 1× binding buffer. After coincubating with 5 μL Annexin V-FITC and 5 μL PI for 15 minutes, apoptosis was detected by flow cytometry (FACSCaliber BD, USA).

### RNA pull-down

Biotin-labeled hsa_circRNA6448-14 probes and control probes were synthesized by Sangon Biotech (Shanghai, China). The lysis buffer included 1 mM PMSF, 100 U RI (RNase Inhibitor, ThermoFisher, EO0384) and 1×PIC (Protease Inhibitor Cocktail, Roche, 04 693 116 001). The cell lysis buffer was incubated with Streptavidin beads (Invitrogen, 65801D, USA) at 25°C for 30 minutes, and the pre-hybridized cell lysates were collected and divided into three groups: beads, antisense, and sense. Beads were washed 5 times with RNA binding buffer, and incubated with miRNA. Finally, TRIzol reagent was added to the beads mixture for RNA extraction, followed by qRT-PCR. The probe sequences are shown in Supplementary Table 2.

### Statistical analysis

Statistical analyses were performed using SPSS 26.0 (SPSS Inc., Chicago, IL, USA) and GraphPad Prism v8.0 (GraphPad Software Inc., San Diego, CA, USA). Data are presented as mean ± standard deviation (SD). The differences between groups were tested using one-way ANOVA, Student’s *t*-test, or chi-square. Receiver operating characteristic (ROC) curves were applied to analyze the diagnostic values of circRNAs. OS and DFS were analyzed with the Kaplan-Meier method and compared by the log rank test. The univariate and multivariate analyses were analyzed by Cox proportional hazards model. Correlations were determined using Pearson’s correlation coefficients. All p values were based on two-sided testing, and p < 0.05 was considered significant.

## Supplementary Material

Supplementary Figures

Supplementary Tables
