# Correction for: hsa_circRNA6448-14 promotes carcinogenesis in esophageal squamous cell carcinoma

**DOI:** 10.18632/aging.104164

**Published:** 2020-09-30

**Authors:** Yaowen Zhang, Xiang Yuan, Ning Yue, Lidong Wang, Junqi Liu, Ningtao Dai, Haijun Yang, Ruitai Fan, Fuyou Zhou

**Affiliations:** 1 Department of Radiation Oncology, Anyang Cancer Hospital, Anyang 455000, China; 2 Department of Radiation Oncology, Henan Key Laboratory for Cancer Research, The first Affiliated Hospital of Zhengzhou University, Zhengzhou 450000, China; 3 Henan Key Laboratory of Cancer Epigenetics, Cancer Institute, The First Affiliated Hospital and College of Clinical Medicine of Henan University of Science and Technology, Luoyang 471000, China; 4 Department of Radiation Oncology, Rutgers - Cancer Institute of New Jersey, Rutgers - Robert Wood Johnson Medical School, New Brunswick, NJ 08903, USA

**Keywords:** correction

**This article has been corrected: **The authors requested to replace Figure 6G that contains a duplicate panel in originally published image. The duplication happened by mistake during the figure processing. This correction has not changed the interpretation or the original conclusions of this work.

The corrected Figure 6G is provided below.

**Figure 6:**
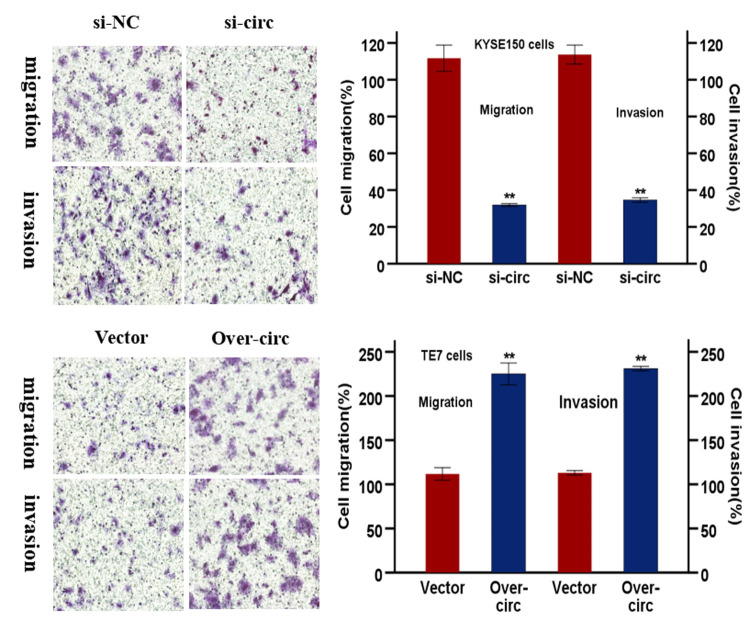
**hsa-circRNA6448-14 promotes ESCC progression in cell lines. (G)** Transwell assays to detect cell migration and invasion capacities of KYSE150 and TE7 cells after transfection. *p < 0.05, **p <0.01.

