# Supplementary material for: hsa_circRNA6448-14 promotes carcinogenesis in esophageal squamous cell carcinoma
Source: Aging (Albany NY). 2020 Aug 15;12(15):15581–602. doi: 10.18632/aging.103650 (PMC7467364; doi:10.18632/aging.103650)
Supplement: Supplementary Figures [file aging-12-103650-s002..pdf]

SUPPLEMENTARY FIGURES

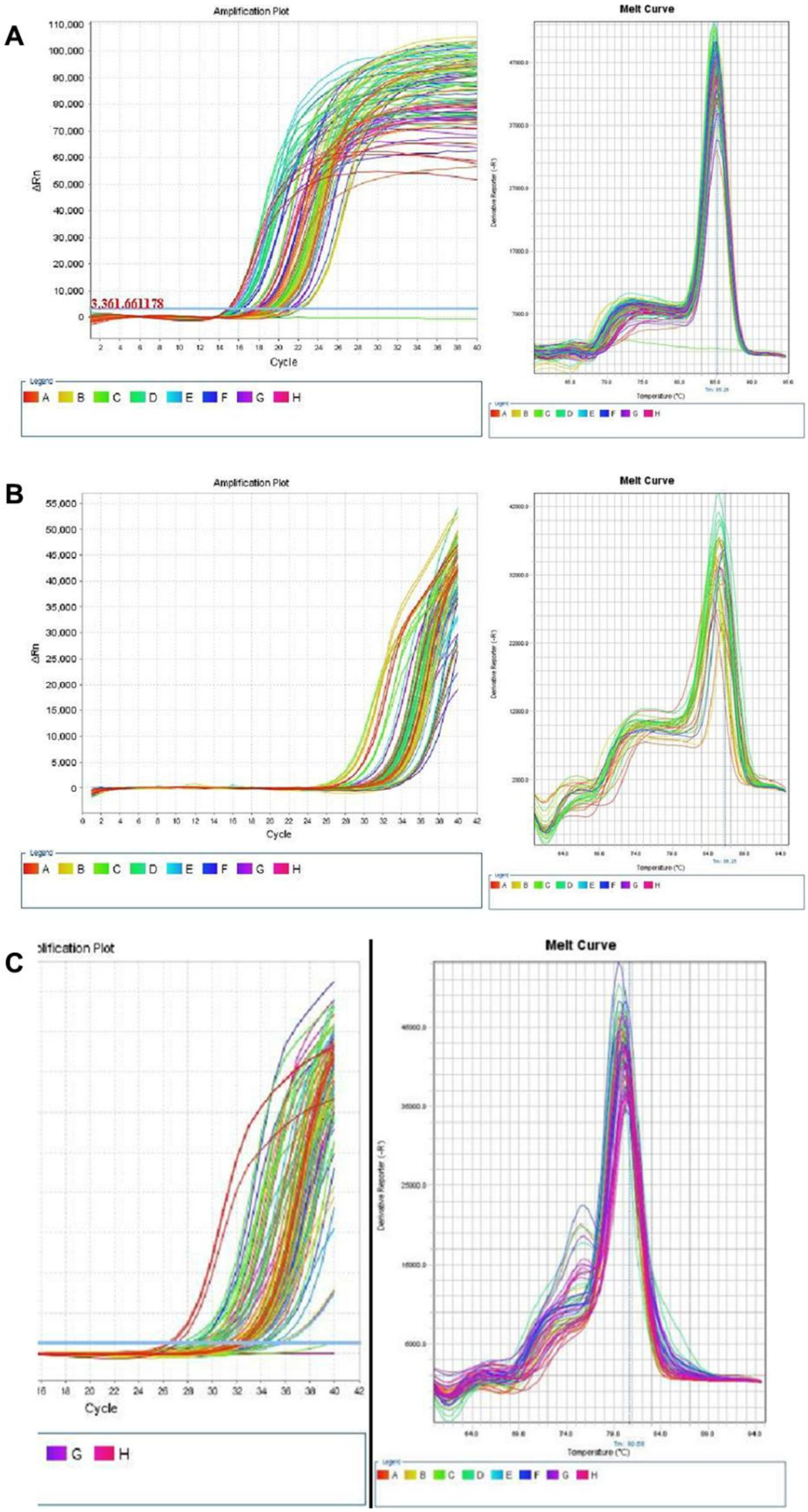

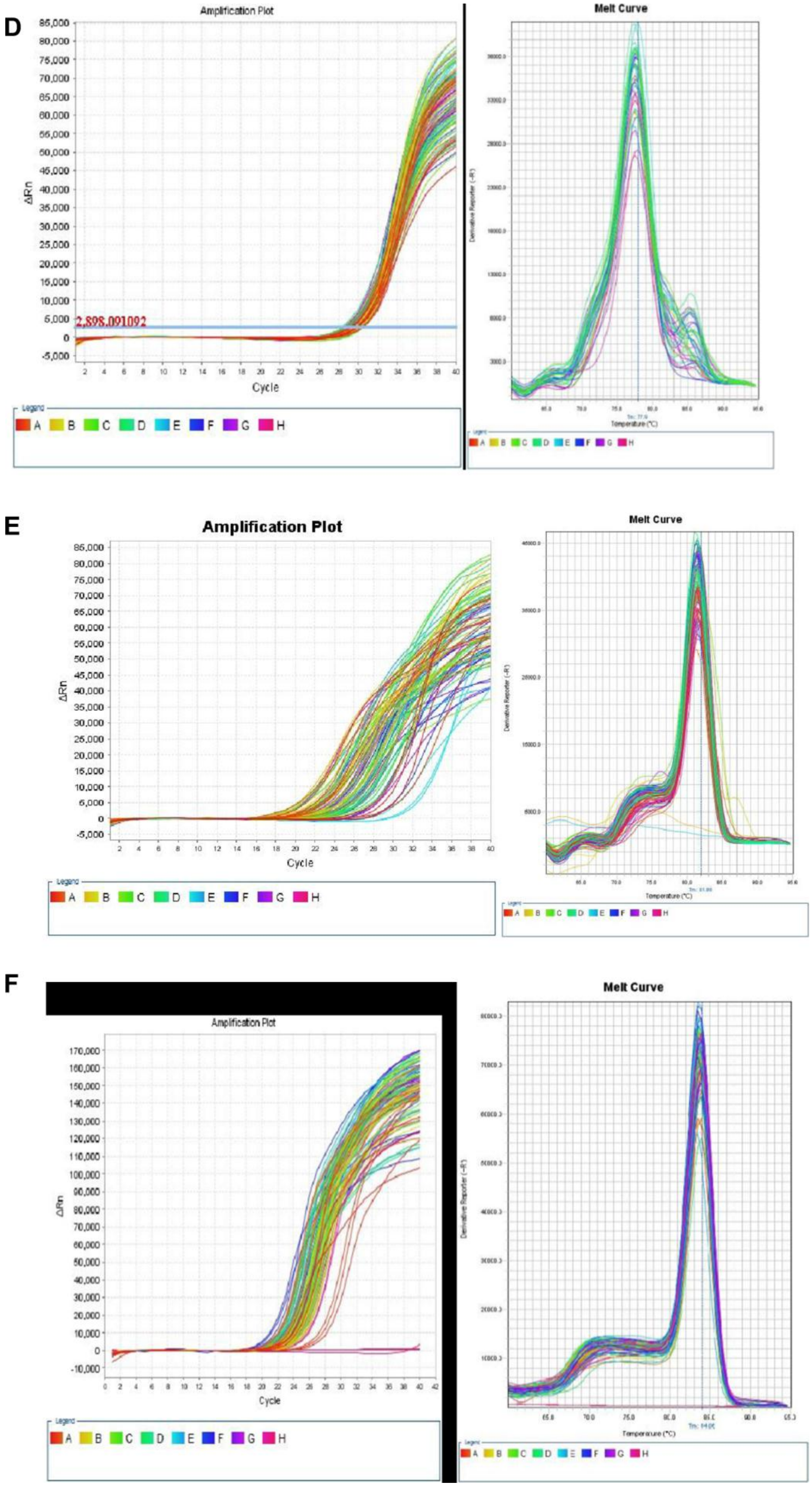

Supplementary Figure 1. qRT-PCR amplification curve and met curve of cirRNAs. (**A**) GAPDH. (**B**) hsa-circRNA6448-14. (**C**) hsa-circRNA15930-8. (**D**) hsa_circ_0110255. (**E**) hsa_circ_0064369. (**F**) hsa_circ_0024108.

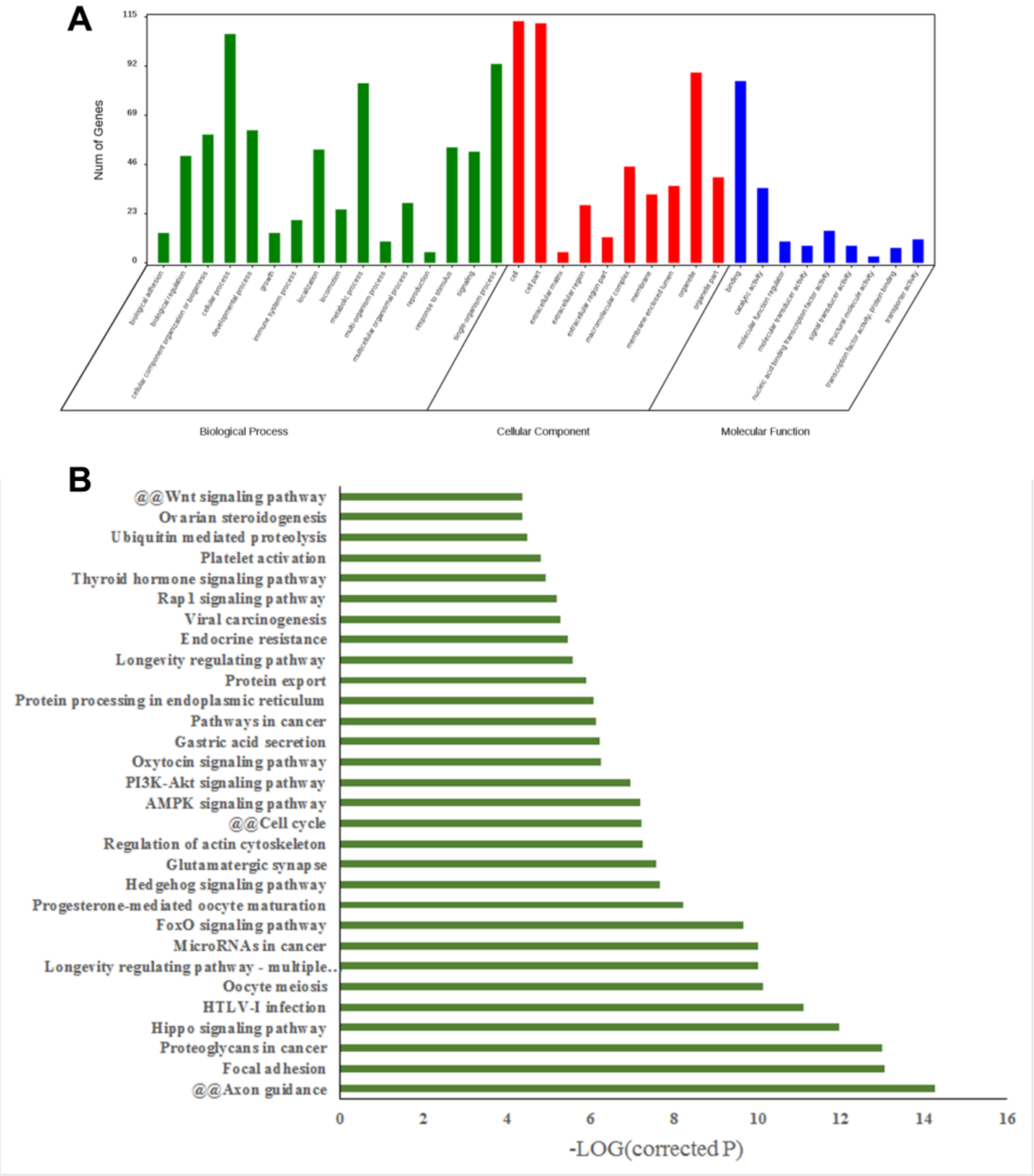

Supplementary Figure 2. Classification of GO function (**A**) and KEGG-Pathway (**B**) of hsa_circRNA6448-14 in ESCC.

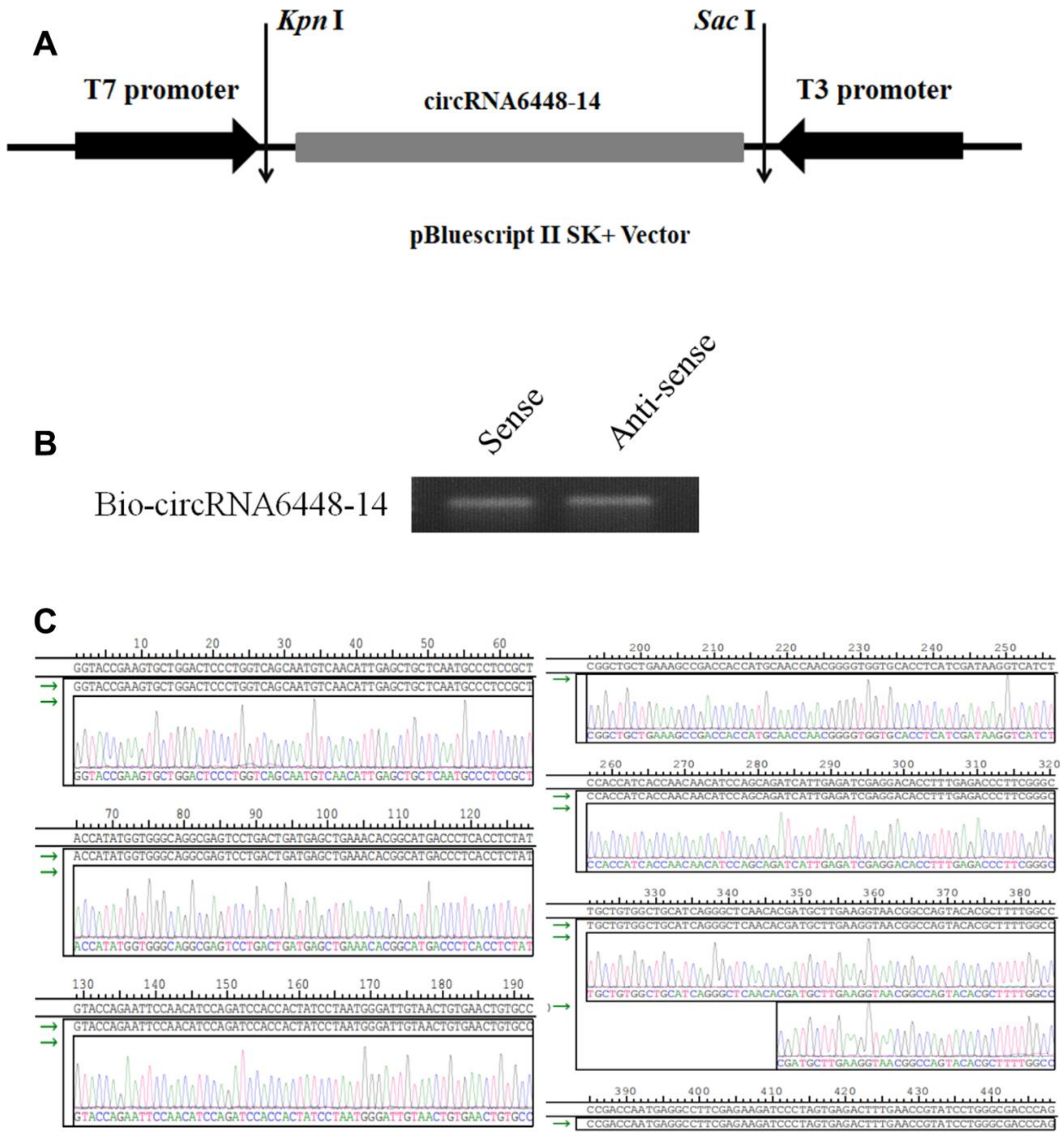

Supplementary Figure 3. hsa_circRNA6448-14 recombinant plasmid. (**A**) schematic diagram of hsa_circRNA6448-14 in vitro transcription template plasmid construction. (**B**) probe agarose gel electrophoresis. (**C**) plasmid sequencing.
