# Supplementary material for: hsa_circRNA6448-14 promotes carcinogenesis in esophageal squamous cell carcinoma
Source: Aging (Albany NY). 2020 Aug 15;12(15):15581–602. doi: 10.18632/aging.103650 (PMC7467364; doi:10.18632/aging.103650)
Supplement: Supplementary Tables [file aging-12-103650-s001..pdf]

SUPPLEMENTARY TABLES

Supplementary Table 1. qRT-PCR primer sequences.

Primer name	Sequences (5' -3')
GAPDH	F: GTGGAGTCCACTGGCGTCT
	R: GTGCAGGAGGCATTGCTGAT
Hsa_circRNA6448-14	F: CCAATGGGGACTGTCATGGA
	R: TCATGCCGTGTTTCAGCTCA
hsa_circ_0110255	F: GCAATCAGCGTGGAGAAGAA
	R: GTACATCAACTGGAGCAGCTCAA
hsa_circRNA15930-8	F: CTTCTGCGCAAGGTCAAGG
	R: TGCGTTGTACCAAGGTCTCC
hsa_circ_0064369	F: GTTGGCATCTACTTCGGACTAGG
	R: CCCATCTTGCTTGTGGAAATC
hsa_circ_0024108	F: CCTGGATAGGCAAGGGATAAC
	R: TCAATGGCATGGTCCACATC

Supplementary Table 2. The probe sequences of five putative target miRNAs.

Primer ID	Sequence (5' -3')
miR-204-5p	RT:CTCAACTGGTGTCGTGGAGTCGGCAATTCAGTTGAGAGGCATAG
miR-455-3p	RT:CTCAACTGGTGTCGTGGAGTCGGCAATTCAGTTGAGGTGTATAT
miR-204-5p	FP:TCGGCAGGTTCCCTTTGTCATC
miR-455-3p	FP:TCGGCAGGGCAGTCCATGGGC
miR	RP:CTCAACTGGTGTCGTGGA

Supplementary Table 3. The information of hsa-circRNA6448-14.

	hsa-circRNA6448-14
Chr	Chr5
Source	Exon5-11
Host gene	TGFBI
Nucleotide Sequence	ctccttgcacgggccggcccagcttccccgcccctggcgtccgctccctcccgctcgcagcttacttaacctggcccgggcggcggaggcgctctcacttccctg<br>gagccgcccgcttgcccgtcggtcgctagctcgctcggtgcgcgtcgtcccgctccatggcgctcttcgtgcggctgctggctctcgccctggctctggccctggg<br>ccccgccgcgaccctggcgggtcccgccaagtcgccctaccagctggtgctgcagcacagcaggctccggggccgccagcacggccccaacgtgtgtgctgt<br>gcagaaggttattggcactaataggaagtacttcaccaactgcaagcagtggtaccaaaggaaaatctgtggcaaatcaacagtcatcagctacgagtgctgtcctg<br>gatatgaaaaggtccctggggagaagggctgtccagcagccctaccactctcaaacctttacgagaccctgggagtcgttggatccaccaccactcagctgtacac<br>ggaccgcacggagaagctgaggcctgagatggaggggcccggcagcttcaccatcttcgcccctagcaacgaggcctgggcctccttgccagctgaagtgctg<br>gactccctggtcagcaatgtcaacattgagctgctcaatgccctccgctaccatatggtgggcaggcgagtcctgactgatgagctgaaacacggcatgaccctca<br>cctctatgtaccagaattccaacatccagatccaccactatcctaatgggattgtaactgtgaactgtgcccggctgctgaaagccgaccaccatgcaaccaacggg<br>gtggtgcacctcatcgataaggtcatctccaccatcaccaacaacatccagcagatcattgagatcgaggacacctttgagacccttcgggctgctgtggctgcatc<br>agggctcaacacgatgcttgaaggtaacggccagtacacgcttttggccccgaccaatgaggccttcgagaagatccctagtgagactttgaaccgtatcctgggc<br>gacccagaagccctgagagacctgctgaacaaccacatcttgaagtcagctatgtgtgctgaagccatcgttgcggggctgtctgtagagaccctggagggcacg<br>acactggaggtgggctgcagcggggacatgctcactatcaacgggaaggcgatcatctccaataaagacatcctagccaccaacggggtgatccactacattgat<br>gagctactcatcccagactcagccaagacactatttgaattggctgcagagtctgatgtgtccacagccattgaccttttcagacaagccggcctcggcaatcatctct<br>ctggaagtgagcggttgaccctcctggctcccctgaattctgtattcaaagatggaacccctccaattgatgcccatacaaggaatttgcttcggaaccacataattaa<br>agaccagctggcctctaagtatctgtaccatggacagaccctggaaactctgggcggcaaaaaactgagagtttttgtttatcgtaatagcctctgcattgagaacag<br>ctgcatcgcggcccacgacaagagggggaggtacgggaccctgttcacgatggaccgggtgctgacccccccaatggggactgtcatggatgtcctgaaggga<br>gacaatcgctttagcatgctggtagctgccatccagtctgcaggactgacggagaccctcaaccgggaaggagtctacacagtctttgctcccacaaatgaagcctt<br>ccgagccctgccaccaagagaacggagcagactcttgggagatgccaaggaacttgccaacatcctgaaataccacattggtgatgaaatcctggttagcggagg<br>catcggggccctggtgcggctaaagtctctccaaggtgacaagctggaagtcagcttgaaaaacaatgtggtgagtgtcaacaaggagcctgttgccgagcctga<br>catcatggccacaaatggcgtggtccatgtcatcaccaatgttctgcagcctccagccaacagacctcaggaaagaggggatgaacttgcagactctgcgcttgag<br>atcttcaaacaagcatcagcgttttccagggcttcccagaggtctgtgcgactagcccctgtctatcaaaagttattagagaggatgaagcattagcttgaagcactac<br>aggaggaatgcaccacggcagctctccgccaatttctctcagatttccacagagactgtttgaatgttttcaaaaccaagtatcacactttaatgtacatgggccgcac<br>cataatgagatgtgagccttgtgcatgtgggggaggagggagagagatgtactttttaaatcatgttccccctaaacatggctgttaacccactgcatgcagaaacttg<br>gatgtcactgcctgacattcacttccagagaggacctatcccaaatgtggaattgactgcctatgccaagtccctggaaaaggagcttcagtattgtggggctcataa<br>aacatgaatcaagcaatccagcctcatgggaagtcctggcacagtttttgtaaagcccttgcacagctggagaaatggcatcattataagctatgagttgaaatgttct<br>gtcaaatgtgtctcacatctacacgtggcttggaggcttttatggggccctgtccaggtagaaaagaaatggtatgtagagcttagatttccctattgtgacagagccat<br>ggtgtgtttgtaataataaaaccaaagaaacata
